# ECTyper: *in silico Escherichia coli* serotype and species prediction from raw and assembled whole-genome sequence data

**DOI:** 10.1099/mgen.0.000728

**Published:** 2021-12-03

**Authors:** Kyrylo Bessonov, Chad Laing, James Robertson, Irene Yong, Kim Ziebell, Victor P. J. Gannon, Anil Nichani, Gitanjali Arya, John H. E. Nash, Sara Christianson

**Affiliations:** ^1^​ National Microbiology Laboratory, Public Health Agency of Canada, Guelph, ON, Canada; ^2^​ National Centre for Animal Diseases, Canadian Food Inspection Agency, Lethbridge, Canada; ^3^​ National Microbiology Laboratory, Public Health Agency of Canada, Lethbridge, AB, Canada; ^4^​ National Microbiology Laboratory, Public Health Agency of Canada, Toronto, ON, Canada; ^5^​ National Microbiology Laboratory, Public Health Agency of Canada, Winnipeg, MB, Canada

**Keywords:** enteric pathogens, *E. coli*, *in silico* serotyping, public health, serotyping

## Abstract

*
Escherichia coli* is a priority foodborne pathogen of public health concern and phenotypic serotyping provides critical information for surveillance and outbreak detection activities. Public health and food safety laboratories are increasingly adopting whole-genome sequencing (WGS) for characterizing pathogens, but it is imperative to maintain serotype designations in order to minimize disruptions to existing public health workflows. Multiple *in silico* tools have been developed for predicting serotypes from WGS data, including SRST2, SerotypeFinder and EToKi EBEis, but these tools were not designed with the specific requirements of diagnostic laboratories, which include: speciation, input data flexibility (fasta/fastq), quality control information and easily interpretable results. To address these specific requirements, we developed ECTyper (https://github.com/phac-nml/ecoli_serotyping) for performing both speciation within *Escherichia* and *Shigella *, and *in silico* serotype prediction. We compared the serotype prediction performance of each tool on a newly sequenced panel of 185 isolates with confirmed phenotypic serotype information. We found that all tools were highly concordant, with 92–97 % for O-antigens and 98–100 % for H-antigens, and ECTyper having the highest rate of concordance. We extended the benchmarking to a large panel of 6954 publicly available *E. coli* genomes to assess the performance of the tools on a more diverse dataset. On the public data, there was a considerable drop in concordance, with 75–91 % for O-antigens and 62–90 % for H-antigens, and ECTyper and SerotypeFinder being the most concordant. This study highlights that *in silico* predictions show high concordance with phenotypic serotyping results, but there are notable differences in tool performance. ECTyper provides highly accurate and sensitive *in silico* serotype predictions, in addition to speciation, and is designed to be easily incorporated into bioinformatic workflows.

## Data Summary

Raw sequence data for the 185 newly sequenced isolates have been deposited under NCBI BioProject PRJNA670237. The ECTyper source code and database files are accessible from the GitHub repository https://github.com/phac-nml/ecoli_serotyping and it is installable through conda and pip. Web-based wrappers are available for Galaxy (https://toolshed.g2.bx.psu.edu/repository?repository_id=677aeba29ae3ba88) and IRIDA (https://github.com/phac-nml/irida-plugin-ectyper). Five supplementary tables are available with the online version of this article.

**Impact Statement**

We developed and validated a new *E. coli in silico* serotype prediction tool, ECTyper, that provides easily interpretable results that are tailored to be incorporated into routine public health laboratory workflows. The tool provides both command-line and web-based interfaces (Galaxy Project [50], IRIDA [35]) allowing for flexible and easy integration into existing pipelines. ECTyper provides highly accurate serotype predictions, even for low-quality whole-genome sequencing (WGS) data for both assembled and raw sequence data, in addition to species resolution within *Escherichia* and *Shigella *. The reported results are designed to be readily interpreted by laboratory staff with clear quality control information. ECTyper and SerotypeFinder both provided highly concordant results with phenotypic serotyping on both a newly sequenced panel of 185 isolates and a large public dataset of 6954 isolates. Based on this validation work, ECTyper can be implemented into public health laboratories with confidence.

## Introduction

*Escherichia* coli is a Gram-negative rod-shaped bacterium that is frequently found in the intestines of many animals, including humans. Most *E. coli* are naturally occurring commensal intestinal flora, but some can be pathogenic, depending on their host and route of infection, and are usually transmitted via the faecal–oral route, leading to foodborne outbreaks in humans. Foodborne illness is a significant global health and economic burden according to the World Health Organization (WHO) [1], with an estimated 600 million annual cases of foodborne illnesses and 420 000 deaths, of which 63 000 are caused by *E. coli* [1, 2]. In Canada, the annual estimates suggest that there are approximately 4 million cases of foodborne illnesses every year, with ~40 000 caused by *E. coli* [3, 4]. There have been numerous revisions to the taxonomy within the genus *Escherichia *, and currently there are five formally named species, *E. albertii *, *E. coli *, *E. fergusonii *, *E. hermanni*, *E. marmotae* and *E. ruysiae *, in addition to six cryptic clades (I–VI) [5–7]. Delineation between *Escherichia* species is difficult due to their high level of genetic relatedness, along with unstable biochemical phenotypes, with *E. coli *, *E. albertii, E. fergusonii* and *Shigella* spp. being the most clinically relevant [8, 9]. As a first step, diagnostic laboratories perform speciation using biochemical assays such as Vitek (bioMérieux, Inc., Durham, NC, USA) and mass spectrometry. Cryptic *Escherichia* lineages are phenotypically indistinguishable from *E. coli *, but can be identified by several multi-locus sequence typing (MLST) schemas [10], ribosomal [11], core-genome MLST [12] and Clermont *et al.* phylotyping [13, 14]. *Shigella* is closely related to *Escherichia *, which poses issues for the identification of samples, and there has been some controversy as to whether they should be merged [15]. The major species of *Shigella* fall into three clusters within *E. coli* lineages, including *S. dysenteriae* types 1, 8 and 10, *S. boydii* and *S. sonnei* [16]. However, diagnostic differentiation between *Escherichia* and *Shigella* species is important from a clinical and epidemiological perspective, as *Shigella* spp. are distinctly different clinical and epidemiological pathogens that can cause dysentery (shigellosis) [17].

The gold standard within-species classification for *E. coli* is phenotypic serotyping with antibodies targeting specific surface somatic (O) and flagellar (H) and, occasionally, the capsular (K) antigens [18, 19]. Rapid, accurate and cost-effective serotyping has long played a vital role in surveillance and outbreak detection activities since it provides crucial subtyping information quickly [20]. Increasingly, whole-genome sequencing (WGS) is being adopted by public health and food safety laboratories to replace traditional methods of strain characterization for surveillance and outbreak response. As more laboratories adapt their operations to utilize WGS within a public health and food safety context, it is important to maintain the ability to derive serotypes from WGS data, in order to leverage existing knowledge and minimize disruptions to ongoing public health surveillance activities.

Phenotypic serotyping methods are laborious, time-consuming and require the maintenance of large libraries of antisera targeting each antigen, and the results are strongly affected by the quality of the antisera and the experience of the technician. There are 181 recognized O-antigens that form part of the large lipopolysaccharides (LPS) structure that is present on the surface of Gram-negative bacteria and is structurally composed of oligosaccharide repeats made up of 3 to 5 sugars [21]. The chromosomal O-antigen gene cluster consists of multiple genes and is responsible for precursor synthesis, transport, assembly and maturation of these precursors to form part of LPS [22, 23]. There are four genes that make ideal biomarkers for predicting O-antigen type due to the key roles that they play in determining the O-antigen structure, which include a flippase (*wzx*), a polymerase (*wzy*) and ABC-dependent transporters (*wzm*/*wzt)* [23]. Interestingly, Ooka *et al.* identified 20 O-antigens shared between *E.albertii* and *E. coli* [24]. Suitable biomarkers for predicting the H-antigen type are primarily based on *fliC*, in addition to *flkA, fllA* and *flmA*, which have been shown to influence some H-antigen types [25]. Phenotypic typing of H-antigens typically takes 2 h, but issues with sample motility and the requirement for several rounds of agglutination reactions can easily increase the time taken for determining serotype to several days if repeat passages are necessary [26, 27]. A benefit of *in silico* typing is that there is no need to go through the time-consuming process of motility induction, since the results are based on the presence of the specific sequences [28, 29].

Multiple tools have been developed for *in silico* serotyping of *E. coli* samples, which predict serotype by identification of closest matching alleles within curated databases of antigen-specific alleles. Three commonly used tools are: SRST2 [30], SerotypeFinder [31] and EToKi EBEis (EnteroBase *Escherichia in silico* serotyping module from EnteroBase Tool Kit) [32]. Both O and H-antigens have variants where the corresponding genes are highly similar and these alleles with high nucleotide identity can pose issues for similarity search-based antigen determination [31]. For example, O17, O44, O73, O77 and O106 are antigens where the alleles of *wzx* and *wzy* have been shown to have greater than 99 % similarity [31] and in the case of H antigens there is high similarity (97–99 %) for H4 and H17 in *fliC*. WGS data can be leveraged to identify the species of an isolate by identifying the closest genome in a curated database such as RefSeq [33]. Tools for performing rapid whole-genome genetic distance comparisons include MASH, which enables high compression of the database and rapid genetic distance calculations [34]. Due to the taxonomic complexity within and between *Escherichia* and *Shigella *, it is important to confirm the species identification of a sample, but none of the existing *in silico* serotyping tools provide this functionality.

Here we present ECTyper, a Python 3 tool for performing *in silico* serotyping and species identification using either assembled or raw sequence data, which was developed as a command-line tool that is web-accessible through Galaxy Project (https://usegalaxy.eu/root?tool_id=ectyper) [1] and IRIDA (https://github.com/phac-nml/irida-plugin-ectyper) [35]. The serotype reports produced by ECTyper are designed to be readily interpretable with QC information for technical staff. We benchmarked the performance of ECTyper along with SRST2, SerotypeFinder, EToKi EBEis on both on a verified dataset of newly sequenced strains and a large collection of publicly available *E. coli* strains.

## Methods

### Development of biomarker database

Sequence similarity-based *in silico* serotyping depends heavily on accurate and comprehensive coverage of different antigen alleles to provide reliable identification results. As a starting point, the allele databases from SRST2 (release date: 30 July 2019) and SerotypeFinder (release date: 28 January 2019) were combined and analysed using db-check v. 0.1.4 (https://github.com/andersgs/db-check) to determine consistency in the antigen assignment for each allele sequence, as well as to remove duplicated and truncated alleles. A total of 8125 *E. coli* samples were downloaded from EnteroBase (10 January 2019) that had either partial or complete serotype information reported on the record, i.e. O157:H7, O157, or H7. The reported O- and H-antigens in the EnteroBase metadata were standardized in accordance with the accepted list of O- and H-antigens at the Canadian National Microbiology Laboratory. Rough, non-motile or unreported antigens were all treated the same with the antigen labelled as ‘-’. To increase representation of diverse alleles for highly prevalent serotypes such as O157, we selected 556 samples from the EnteroBase dataset (Table S1, available in the online version of this article). Additionally, to further improve the representation of O77, H28 and H52 antigens, three alleles were extracted from GenBank NCBI (AB972416.1, JH965342 and AVRH01000047). blastn v. 2.7.1 was used to query the initial set of allele sequences from SRST2 and SerotypeFinder with a requirement of >=97 % coverage and identity. Novel alleles from EnteroBase were excluded from the database if they did not possess a stop codon at the 3′ end. Another round of deduplication and allele assignment consistency was performed with db-check v. 0.1.4 with the addition of the new alleles from EnteroBase.

Iguchi *et al.* identified 16 high-similarity O-antigen similarity groups [36] and we replicated this work by using ClustalW v. 2.1 [37] to align alleles within each gene and pairwise distances were calculated for all alleles using ape [38] (Table S2). For O-antigen biomarker genes the clustering threshold was set at >=98 % identity and coverage and for H-antigen genes it was set to >=99 %. From the perspective of the clinical laboratory, it was preferred to have ambiguity in a result requiring further confirmation rather than a fully resolved and incorrect result. For this reason, O-antigens that form a high-similarity group are all reported as members of the group unless there is >1 % divergence in the antigen allele hits. For example, the O2 and O50 are closely related and are reported as O2/O50 when the blastn hits are >99 % identical.

The minimum thresholds for coverage and identity that are necessary to unambiguously identify each antigen cluster were determined by computing all-against-all blastn of all alleles within each gene. The threshold was assigned to be the level of coverage and identity where all of the hits correspond to a single antigen cluster with a lower bound of 1 % coverage in cases where all hits from a query match to the same antigen. This is useful in cases where the sequence coverage of the marker genes is poor but the matching sequence identified is highly diagnostic for the antigen allele. For example, O88-4-*wzx* allele, a member of the O88 serogroup, has no hits outside the O88 serogroup, thus a default minimum threshold of 1 % coverage and 90 % identity are assigned. Otherwise, if a given query sequence had hits to multiple different antigens then the minimum coverage and identity thresholds for that antigen were increased to the level that would unambiguously identify it as a single antigen cluster. This information is used to set individual antigen thresholds for both coverage and identity based on its uniqueness.

### Theory and implementation

**Fig. 1.**
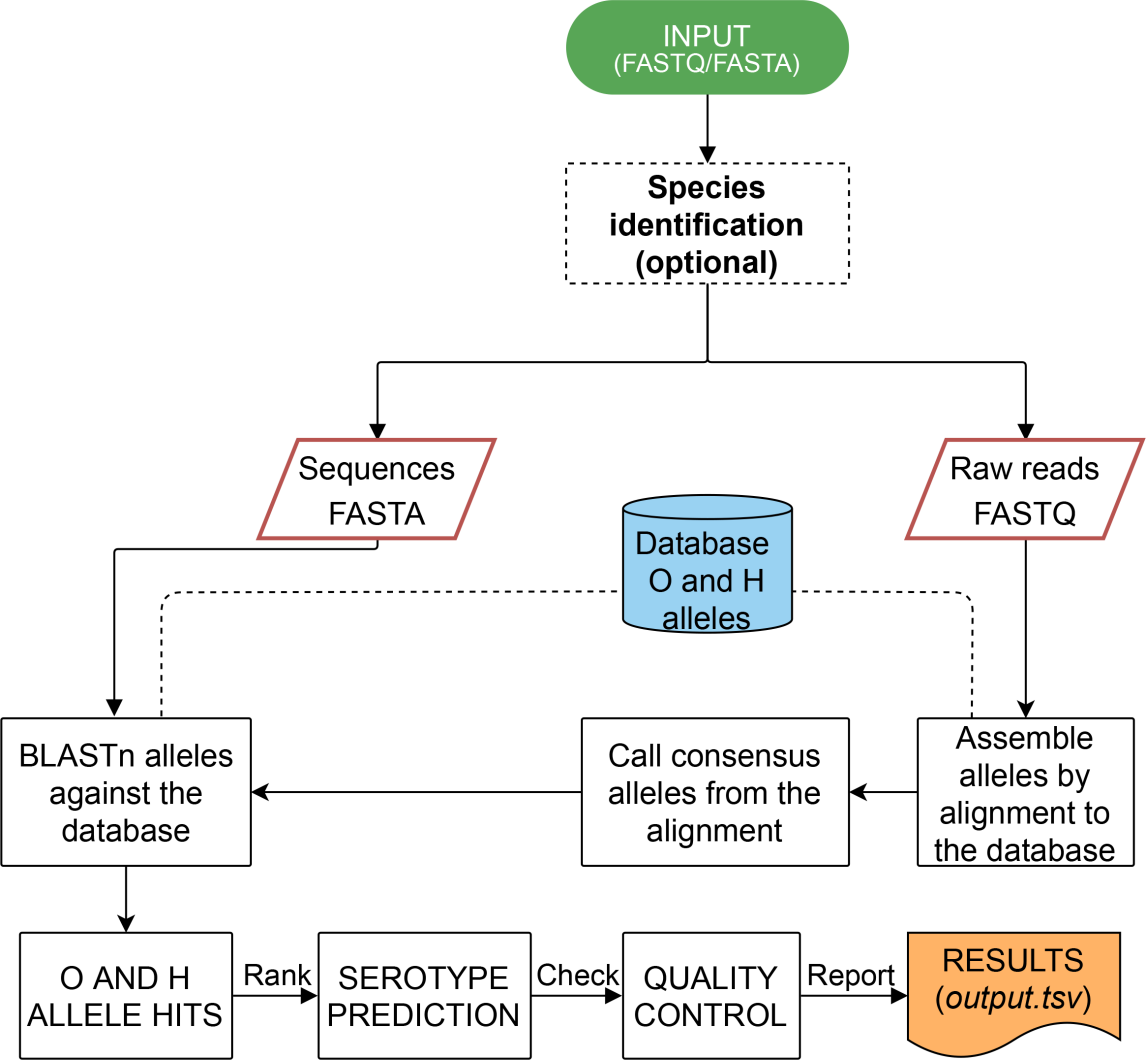
Flowchart outlining the major stages within *ECTyper*. Input can be either raw reads or assemblies. Species identification is performed if the ‘--verify’ parameter is specified using MASH to determine the closest representative genome in NCBI RefSeq. Antigen predictions only proceed if the species is *E. coli.* In the case of raw reads as input, there is a preprocessing stage that aligns the reads against curated databases of genes used to predict O- and H-antigens and produce a consensus sequence. After the preprocessing stage, both reads and assemblies are processed the same. The best matching alleles for each of the genes is identified using blastn based on both *%*identity and %coverage values. A final report is output in tab-delimited format with the summary QC values (Table 1). See the Methods section for further details.

The ECTyper software is designed to fit into the workflows of reference and diagnostic laboratories performing typing of *E. coli* samples by performing both speciation within *Escherichia* and *Shigella* and *in silico* serotyping. ECTyper requires minimal user-specified parameters and accepts both assembled genomes and raw Illumina sequence data, which provides flexibility to the user, depending on their bioinformatic workflows. The ECTyper workflow consists of an optional species identification step using the RefSeq database of curated genomes and species assignments, followed by identification of closest matching alleles of the key genes for determining O- and H-antigens (Fig. 1). ECTyper requires accurate species designations in its database and it is known that there are inconsistencies in RefSeq with the taxonomy of cryptic *Escherichia* clades due to their inconsistent phylogenetic placement, which will hopefully be resolved in future releases [7]. The optional species identification step is performed if the user specifies the ‘--verify’ parameter using MASH v. 2.0.0 [34] against the NCBI RefSeq database. The closest matching genome with the smallest MASH distance is used to predict the species of the sample. If the sample is labelled as anything other than *E. coli *, the software does not proceed with *in silico* serotyping. The results are provided as a tab-delimited file with quality control information for troubleshooting purposes.

**Table 1.** Quality control values and their assignments based on the nine scenarios

Value	Scenario
PASS (REPORTABLE)	Both O and H-antigen alleles meet or exceed both minimum %identity or %coverage individual allele thresholds and a single serogroup is predicted both for O and H
FAIL (-:- TYPING)	A sample is *E. coli* and both O- and H-antigens are not called by the tool. For example,the reported serotype is -:-
WARNING (WRONG SPECIES)	A sample is non-* E. coli* (e.g. *Shigella boydii *) based on the NCBI RefSeq reference assemblies
WARNING MIXED O-TYPE	A mixed O-antigen call is predicted requiring a further wet-lab confirmation (e.g. O17/O77/O73/O106)
WARNING (-:H TYPING)	A sample is *E. coli* and O-antigen is not predicted. For example, reported serotype is -:H18
WARNING (O:- TYPING)	A sample is *E. coli* and H-antigen is not predicted. For example, reported serotype is O17:-
WARNING (O NON-REPORT)	O-antigen alleles do not meet minimum %identity or %coverage thresholds
WARNING (H NON-REPORT)	H-antigen alleles do not meet minimum %identity or %coverage thresholds
WARNING (O and H NON-REPORT)	Both O and H-antigen alleles do not meet individual minimum %identity or %coverage thresholds

The process for identification of antigen sequences differs slightly depending on whether the user is providing assembled or raw sequence data (Fig. 1). For raw sequence inputs, a preprocessing step is performed where reads are aligned to the database of reference O- and H-antigen alleles by Bowtie2 v. 2.4.2 [39] followed by calling the consensus sequence using BCFtools v. 1.8 [40] and seqtk v. 1.3 [41]. Following identification of the consensus sequence, both assembled and raw sequence inputs are processed identically. Using the databases of O- and H-antigen alleles, each genome is queried using blastn [42] and the hits are filtered using default thresholds of >=95 % identity and >=90 % coverage for O-antigen alleles and >=90 % identity and >=50 % coverage for H-antigen alleles. The specific antigen allele is identified by sorting hits to select the allele with the highest coverage and identity. The allele gene score is calculated as a product of %identity and %coverage normalized by the maximum possible product value of 10 000 favouring alleles with the largest %identity and %coverage values (gene score=(%identity*%coverage)/10 000).

To improve the accuracy and resolution of O-antigens, ECTyper bases the overall call for the O-antigen type on the information provided from both *wzx*/*wzy* or *wzm*/*wzt* gene pairs. Candidate O-antigens are scored based on the sum of the scores from both genes in a pair and if a gene is missing it contributes 0 to the score for that O-antigen. Based on *wzx*/*wzy* or *wzm*/*wzt* O-antigen allele pairs, the highest scoring antigen is reported. It is possible for multiple antigens to have highly similar scores due to inherent sequence similarity between them, or sequence quality issues, and in these cases, ECTyper reports all of the candidate antigens that are >99 % identity and 100 % coverage. Cluster memberships of O-antigens with either identical or highly similar *wzx*/*wzy* or *wzm*/*wzt* alleles is presented in Table S2. For some O-antigens, the gene pairs only provide minimal resolution power, with the cumulative differences in *wzx*/*wzy* or *wzm*/*wzt* alleles as low as one SNP: for example, between O118 and O151, or O123 and O186 [31]. In contrast to O-antigen prediction, the H-antigens are predicted based on a single gene, with the highest scoring allele determining the H-antigen prediction. The *fliC*, *flkA*, *fllA*, *flmA* and *flnA* genes are used for H-antigen prediction, with *fliC* being represented by the largest number of alleles in the database.

Reporting requirements for reference and diagnostic laboratories frequently require quality information to determine if the result meets their criteria for reporting to clients. To address this need, ECTyper has a built-in quality reporting module that provides clear quality control flags (Table 1) along with required information for diagnosing issues, with the result based on extensive consultation with diagnostic laboratories within the Canadian National Microbiology Laboratory. The main QC criteria include species check, presence and absence of the O- and H-antigen alleles, quality of the antigen prediction based on individual allele %identity and %coverage thresholds, and distance to alleles of other antigens (Table 1). These QC flags allow for quick sample screening in a diagnostic setting and ensure that the sample meets the minimum reporting requirements and is a feature that is unique to ECTyper compared to other *E. coli in silico* typing tools. Samples that are assigned a PASS flag would be readily reportable due to the presence of close database matches for each of the antigen genes with adequate coverage to make a high-quality serotype prediction. Samples that are flagged as anything other than PASS should be reviewed in more detail by the technician to ascertain what troubleshooting steps are necessary. Some situations requiring troubleshooting include non-* E. coli *, potentially contaminated samples and low-quality WGS data resulting in low-quality database matches.

### Construction of benchmarking datasets

The first dataset is a collection of 185 newly sequenced isolates with verified phenotypic serotype information representing 122 diverse serotypes (Table S3) where any discrepancies in predictions could be verified with additional phenotypic testing. The second dataset consisted of a broad collection of publicly available genomes to assess the performance of the tools on a diverse array of serotypes contributed from different laboratories (Table S4). Reads for these genomes were downloaded from the Sequence Read Archive and assembled using shovill and each of the genomes was assessed for quality and completeness using Quast and CheckM as described above. EnteroBase performs quality assessment of genomes before inclusion into their database, but there is the possibility that the read sets changed since they were analysed and we identified seven genomes that failed to assemble. Species confirmation of the candidate *E. coli* genomes was performed using MASH within ECTyper and the RefSeq database. Of the original 8125 genomes we removed any genomes in the construction of the allele databases in addition to any genomes that failed assembly checks, which yielded 6954 genomes for the public benchmarking dataset (Table S4).

### *In silico* serotyping tools benchmarking

The performance of ECTyper v. 1.0.0, along with three existing tools, SerotypeFinder v. 2.0.1 (released: 28 January 2019), EToKi EBEis v. 1.0 (released: 1 December 2019) and SRST2 v. 0.2.0 (released: 30 July 2019) was compared using the two different datasets described above using the default parameters for each tool. SRST2 requires raw reads as input and does not have an assembly-based mode. EToKi EBEis, on the other hand, only supports assembly as input. Both SerotypeFinder and ECTyper can utilize raw and assembled data.

Concordance of *in silico* serotype predictions was measured independently using the O- and H-antigens for each sample where a valid antigen was reported. Since *in silico* predictions will not necessarily reflect phenotype, we did not measure concordance for antigens that were reported as rough (Table S4). The predicted antigen determined by each tool was compared against the laboratory-reported antigen and the results were categorized into five types: (1) perfect match (PM); (2) ambiguous match (AM); (3) incorrect prediction (IP); (4) no prediction (NP); (5) not reported (NR). PMs required the reported and *in silico* predicted serotype to be the same. AMs were allowed when the reported antigen was contained in the list of potential antigens reported by a tool in a mixed call, for example, if a sample was reported to be O2 for the O-antigen, but predicted to be O2/O50. IPs were cases where the tool predicts an antigen that does not match any of the reported antigen(s). Samples where a tool did not issue an antigen prediction were listed as NP. NR samples include rough, missing and non-motile assignments and these cases were not analysed further.

### Phenotypic serotyping

A panel of 185 isolates representing a diverse collection of serotypes with high confidence in their assignments. Somatic (O) and flagellar (H) antigens were identified by standard agglutination methods for identification of O1 to O188 and H1 to H56 following the Edwards and Ewing serotyping protocol [18] using commercially available antisera from SSI Diagnostica (Copenhagen, Denmark) by the accredited serotyping laboratory within the Canadian National Microbiology Laboratory.

### WGS

Overnight cultures of *E. coli* were grown in BHI broth and genomic DNA extraction was extracted using the Qiagen EZ1 biorobot using the EZ1 DNA tissue kit. Sequencing libraries were prepared using Illumina Nextera XT library preparation kit and were sequenced on Illumina MiSeq using the MiSeq 600 cycle reagent version 3 kit according to standard manufacturer’s protocols.

### Genome assembly and quality metrics

The Illumina paired reads from both the Guelph Reference Services and EnteroBase public datasets were both assembled using the shovill v. 1.1.0 pipeline (https://github.com/tseemann/shovill) [43] with the following parameters: --gsize 5000000 --assembler spades --trim --depth 0 --mincov 0 --minlen 0. The quality and completeness of the assemblies were assessed using QUAST v. 5.0.2 [44] and CheckM v. 1.1.3 [45], respectively. Genome assemblies were excluded that had a size <4 MB or >6.5 MB, N50 <20 KB or a CheckM completeness score <95.

## Results and discussion

### Construction of the ECTyper allele database

A combined dataset of antigen alleles was constructed using the databases from SRST2 (524 alleles), SerotypeFinder (60 alleles) and NCBI (3 alleles). Additionally, we extracted a total of 936 O- and H-antigen alleles from 556 EnteroBase genomes (Table S1). These genomes were not included in the subsequent benchmarking experiments using the public data. The resulting ECTyper database v. 1.0.0 contains a total of 1523 alleles representing 179 O- and 53 H-antigens, respectively. Specifically, O-antigens are represented by 505 *wzx*, 570 *wzy*, 41 *wzm* and 40 *wzt* alleles, while H-antigens are represented by by 349 *fliC*, 11 *flkA*, 4 *fllA*, 2 f*lmA* and 1 *flnA* alleles.

Since it is known that there is a high degree of sequence similarity between some antigen-coding alleles [36], we determined the pairwise sequence similarity of each of the biomarker alleles in the ECTyper database and identified high-sequence-similarity clusters. We identified 16 clusters of highly similar O groups which represent 35 O-antigens, in addition to 2 H groups consisting of 4 individual H-antigens (Table S2). Each of the flagellar clusters H4/H17 and H1/H12 only differ by one nucleotide difference (Table S2). ECTyper reports all potential antigens when the top match is localized to an O-antigen high-sequence-similarity cluster and all hits are >99 % identical. This ambiguity in reporting is desirable from a diagnostic laboratory perspective, since it is preferable to only report what is a high confidence result.

### Newly sequenced dataset benchmarking

The *in silico* predictions for ECTyper, SerotypeFinder, SRST2 and EToKi EBEis were classified into the five categories described in the Methods section for the 185 newly sequenced isolates with confirmed serotype information using Illumina paired-end sequencing (Table 2). For O-antigens, SRST2 was found to have the highest number of samples with fully matching predictions with 169 (PM) but also had the highest number of incorrect predictions with 14 (IP) (Table 2). When using raw data, ECTyper had the second highest O-antigen matches (PM) with 163 samples and instead of incorrect predictions, there was a higher number of ambiguous results (AM) with 16 (Table 2). ECTyper identified 22 discordant isolates (16 AM and 6 NP) selected for further analysis (Table S5). The primary cause was the inability to distinguish the O-antigens that belong to high-similarity groups [36]. Using assemblies as input, ECTyper had the highest concordance with phenotypic serotyping for this dataset (96.8 % O, 100 % H) followed by EToKi (94.6 % O, 98.4 % H) and SerotypeFinder (93.5 % O, 99.5 % H) antigen typing concordance (Table 2). There was a minimal difference when using reads as input with ECTyper, which resulted in a slight increase in ambiguous (AM) results (Table 2). EToKi EBEis had almost double the number of ambiguous results compared to SerotypeFinder and ECTyper (Table 2). All four tools were able to provide a perfect match for 78–91 % of samples and fully concordant O-antigen predictions (PM and AM) for 91–96 % of the 185 samples and only a single incorrect prediction by one tool. This demonstrates that *in silico* predictions based on marker genes are highly accurate.

**Table 2.** *In silico* serotype prediction benchmarking on newly sequenced isolates. A total of 185 *E. coli* isolates with complete serotype information for both O- and H-antigens were used to benchmark the performance of four *in silico* prediction tools (Table S3)

	O-antigen				H-antigen			
	Perfect match (PM)	Ambiguous match (AM)	Incorrect prediction (IP)	No *in silico* prediction (NP)	Perfect match (PM)	Ambiguous match (AM)	Incorrect prediction (IP)	No *in silico* prediction (NP)
ECTyper (assembly)	163	16	0	6	185	0	0	0
ECTyper (reads)	163	14	1	7	185	0	0	0
SerotypeFinder	154	19	0	12	183	1	0	1
EToKi EBEis	145	30	2	8	182	0	0	3
SRST2	169	0	14	2	181	0	1	3

SRST2 had the fewest number of samples with no serotype prediction (NP) for O-antigens, while SerotypeFinder had the highest. ECTyper clearly benefited from the expanded allele set, since its performance was in-between SRST2 and SerotypeFinder (Table 2). The differences between ECTyper and SRST2 are likely due to algorithm differences, since ECTyper is built on the database from SRST2. However, there was only a minor benefit to the expanded allele database for H-antigens compared to SerotypeFinder and SRST2 (Table 2). H-antigen predictions were all highly accurate and showed >97 % concordance with phenotypic serotyping for the four tested tools (Table 2). O-antigen marker genes had higher rates of no matches (NP) compared to H-antigens (Table 2). This could be due in part to sequence compositional differences in the respective genes, which can cause low sequencing coverage, as has been described previously for *Salmonella *, where low GC content in *wzx* and *wzy* loci (25–35 %) causes these regions to have low sequencing depth when libraries are prepared using the Nextera XT kit [46, 47]. Overall, ECTyper strikes a good balance between sensitivity and specificity for *in silico* predictions of O- and H-antigens, but all of the tools tested produced reasonably accurate antigen predictions for the majority of the tested isolates in this panel.

### EnteroBase public dataset benchmarking

The benchmarking of the four *in silico* serotyping tools was extended using a large dataset of 6954 publicly available *E. coli* samples from EnteroBase which contained 187 distinct O- and 53 H-antigens. The O- and H-antigens were analysed independently, since both antigens were not always reported. There were 6905 samples with a reported O-antigen and 3722 samples with a reported H-antigen that were used for tool benchmarking (Table 3). The number of samples that failed to produce an O-antigen prediction (NP) remained low for ECTyper, EToKi EBEis and SerotypeFinder, with <=7 % for the three tools (Table 3). However, there was a larger number of samples failing to produce a predicted serotype using SRST2, with 17 % for O-antigens and 31 % for H-antigens (Table 3). This could be due in part to the use of the raw sequence reads as the basis for the serotype predictions, since the assembly-based input to ECTyper had <1 % of samples without an O-antigen prediction compared to 6 % for read-based input (Table 3). Similarly to the newly sequenced dataset, there were more instances of missing marker genes for O-antigens compared to H-antigens (Table 3).

**Table 3.** *In silico* serotype prediction benchmarking on a large public dataset. A total of 6954 samples with complete or partial serotype information were used to benchmark 4 tools for *in silico* serotype prediction accuracy (Table S4). Since complete antigen information was not available for all samples, there was a total of 6905 samples with O-antigen information and 3722 samples with designated H-antigens

	O-antigen				H-antigen			
	Perfect match (PM)	Ambiguous match (AM)	Incorrect prediction (IP)	No *in silico* prediction (NP)	Perfect match (PM)	Ambiguous match (AM)	Incorrect prediction (IP)	No *in silico* prediction (NP)
ECTyper (assembly)	6071	176	595	63	3309	0	396	17
ECTyper (reads)	5756	149	588	412	3303	0	390	29
SerotypeFinder	5896	262	586	161	3308	25	386	3
EToKi EBEis	5288	572	555	490	3304	7	393	18
SRST2	5125	45	559	1176	2269	12	265	1176

Assembly-based inputs had the highest numbers of PMs for ECTyper (87.9 % O, 88.9 % H) and SerotypeFinder (85.4 % O, 88.9 % H) when compared to the user reported serotypes for this dataset (Table 3). ECTyper performed similarly when using reads as input but there was a 4.5 % decrease in PM compared to assembly-based O-antigen predictions and 0.2 % for H-antigens (Table 3). Both EToKi EBEis (76.6 % O, 88.8 % H) and SRST2 (74.7 % O, 61 % H) had significantly lower numbers of PMs compared to the reported O-antigens (Table 3). However, EToKi EBEis had similar accuracy for H-antigen predictions compared to SerotypeFinder and ECTyper. When ambiguous matches were also taken into account (PMs and AMs), the differences in concordance between reported and predicted serotypes for assembly based inputs are reduced considerably with ECTyper (90.4 % O, 88.9 % H), SerotypeFinder (89.2 %O, 89.6 % H) and EToKi EBEis (84.9 %O, 89 % H). SRST2 (74.9 %O, 61.3 % H) lags behind the other tools, primarily because of the much larger number of failed predictions (NPs) as the numbers of incorrect predictions (IPs) for each tool were very consistent, with an average of 8.3 % for O-antigens and an average of 9.8 % for H-antigens found across the four tools (Table 3). SerotypeFinder and ECTyper were both highly accurate and outperformed SRST2 and EToKi EBEis on this large and heterogeneous multi-source dataset, with ECTyper seeing a minor drop in typing performance on raw unassembled inputs.

### Discordant antigen predictions observed for EnteroBase dataset across all tools

Samples with disagreement between one or more tools were analysed in more detail to understand if there was potentially an issue with the reported serotype information, since it was not possible to phenotypically confirm them. Lack of agreement between reported and predicted serotypes could be due to inaccurate predictions or point to potential errors in the reported serotype. For example, serotyping within *E. coli* is complicated by known cases of antigens with cross-reactivity, one of the pitfalls of phenotypic sub-typing [18, 31, 48]. There were 880 samples that had at least 1 tool report a discordant prediction across either O- and/or H-antigens (Table S4). This represents 13 % of the dataset. This demonstrates that for the vast majority of the samples, *in silico* predictions are highly accurate. Of the 880 samples, there were 100 samples where a single tool produced a discordant result (IP). These are highly likely to be due to issues with the specific tool, instead of an issue with the reported serotype information. SRST2 had the highest number of unique issues, with 35 samples with a conflict in the O-antigen and 9 for the H-antigen, which represents a total of 44 samples. This result is interesting due to the fact that SRST2 is the second largest contributor of alleles to ECTyper database with 21 unique errors across both O- and H-antigens. SerotypeFinder had the lowest number of unique errors, with seven samples. Due to the extensive sharing of alleles from SerotypeFinder and SRST2 with ECTyper it is not unexpected that there would be low numbers of unique errors and these biases could reinforce incorrect allele designations.

There are 780 samples where the predictions from more than 1 tool did not match what was reported in the serotype record (Table S4). These disagreements represented up to 11 % of the dataset and may be the result of numerous factors, including issues with the antigen associated with alleles in each of the databases, metadata errors, or phenotypes that are the result of activity not controlled by the genes used for predictions [21]. The O2 and O50 serotypes are an example of a difference in phenotype that is determined by a factor outside of the genes used to predict the O-antigen. There is a very high similarity between O2 and O50, with their individual alleles demonstrating 99.9 % nucleotide identity [22]. The cause for the difference in their phenotypes is the result of a point mutation in *fdtB*, which results in the absence of d-Fucp3NAc in the O50 antigen [22]. Similarly problematic antigens for sequence similarity predictions based on the defined biomarker databases include O17, O44, O73, O77 and O106 serogroups, which have genetic identity of greater than 99.8%, but glucosyltransferase genes alter their side-chain composition [22]. In addition to cases where there are issues with high genetic similarity, there are instances of clearly incorrect serotype designations in the public dataset. For example, O157 is the most prevalent antigen in the dataset, with 2865 samples and an overall accuracy rate of 97 %. However, there appears to be an issue with the published serotype assignment in 59 cases, where all of the tools predicted an O-antigen other than O157. A further examination of these 59 discrepant samples shows 40 different O-antigens being predicted, none of which belong to a group that is genetically similar to O157. Additionally, there were 42 samples where H17 was predicted to be H4 by multiple tools. As described earlier, H4 and H17 are highly similar genetically, differentiated by a single SNP. These antigens are even difficult to type reliably in the laboratory using agglutination, since there are problems with antisera cross-reactivity [31]. This represents a limitation of all *in silico* tools that utilize sequence similarity-based approaches if there is no specific nucleotide difference needed to specify one type instead of another. However, identification of these mutations is complex and would require laborious phenotypic confirmation, and for many cases, it is not necessary, as laboratories can rely on WGS-based analyses to determine the relatedness of organisms more accurately.

## Conclusion

As WGS becomes standard within public health laboratories, it is important to minimize disruptions to outbreak and surveillance activities by providing *in silico* serotype predictions. ECTyper was designed to meet the specific needs of diagnostic laboratory reporting requirements and provides both species and serotype predictions based on WGS data. The results are provided in a format that is readily interpretable by technicians performing routine typing of samples with clear quality control information. Consistently between both datasets both ECTyper and SerotypeFinder achieved the highest rates of concordance with phenotypic serotyping. Overall, there were high rates of concordance between predicted and reported serotypes based on genes for O- and H-antigens, respectively. However, there are multiple antigens that cannot be identified reliably using the existing biomarkers due to their high genetic similarity. The ability to resolve highly similar serotypes could be improved by the inclusion of cgMLST, or other whole-genome genetic distance-based approaches, as has already been done for *Salmonella* [49]. The inclusion of species resolution within ECTyper and its high accuracy in predicting serotype information from WGS data will make it a highly useful tool for laboratories performing routine testing of *E. coli *.

## Supplementary Data

Supplementary material 1

Supplementary material 2

Supplementary material 3

Supplementary material 4

Supplementary material 5
